# Immunotherapy Strategy for Systemic Autoimmune Diseases: Betting on CAR-T Cells and Antibodies

**DOI:** 10.3390/antib13010010

**Published:** 2024-02-01

**Authors:** Vitaly Chasov, Ekaterina Zmievskaya, Irina Ganeeva, Elvina Gilyazova, Damir Davletshin, Marat Khaliulin, Emmanuel Kabwe, Yuriy N. Davidyuk, Aygul Valiullina, Albert Rizvanov, Emil Bulatov

**Affiliations:** 1 Institute of Fundamental Medicine and Biology, Kazan Federal University, 420008 Kazan, Russia; 2 Division of Medical and Biological Sciences, Tatarstan Academy of Sciences, 420111 Kazan, Russia; 3 Shemyakin-Ovchinnikov Institute of Bioorganic Chemistry, Russian Academy of Sciences, 117997 Moscow, Russia

**Keywords:** immunotherapy, autoimmune diseases, monoclonal antibodies, bispecific antibodies, CAR-T cells

## Abstract

Systemic autoimmune diseases (SAIDs), such as systemic lupus erythematosus (SLE), systemic sclerosis (SSc) and rheumatoid arthritis (RA), are fully related to the unregulated innate and adaptive immune systems involved in their pathogenesis. They have similar pathogenic characteristics, including the interferon signature, loss of tolerance to self-nuclear antigens, and enhanced tissue damage like necrosis and fibrosis. Glucocorticoids and immunosuppressants, which have limited specificity and are prone to tolerance, are used as the first-line therapy. A plethora of novel immunotherapies have been developed, including monoclonal and bispecific antibodies, and other biological agents to target cellular and soluble factors involved in disease pathogenesis, such as B cells, co-stimulatory molecules, cytokines or their receptors, and signaling molecules. Many of these have shown encouraging results in clinical trials. CAR-T cell therapy is considered the most promising technique for curing autoimmune diseases, with recent successes in the treatment of SLE and SSc. Here, we overview novel therapeutic approaches based on CAR-T cells and antibodies for targeting systemic autoimmune diseases.

## 1. Introduction

Approximately 5–10% of the world’s population suffers from autoimmune diseases [1]. In these patients, a highly complex network of cytokines and their receptors on immune cells destroys healthy tissues and becomes overactive. Many various physiological systems, including the skin, joints, kidneys, lungs, heart and blood cells, might be affected by the symptoms of these disorders. Conventional treatment, mainly glucocorticoids and immunosuppressants, has long been the mainstay of therapy for both moderate and severe disease, but has poor results and low specificity [2]. Among patients, there are many who suffer from the side effects of this type of therapy, as they are forced to take medication for many years [3]. It was not until 2011 that the US Food and Drug Administration (FDA) authorized the first biological drug (belimumab) for the treatment of people with active SLE [4].

Particularly for individuals who do not react to traditional therapies, targeted therapy has recently become a more promising option [5]. Novel biological agents with different mechanisms of action, including monoclonal (mAb) and bispecific antibodies (BsAb), can target B cells, co-stimulatory molecules, and cytokines or their receptors, demonstrating their clinical efficacy and safety [6,7]. Some agents, such as rituximab, tocilizumab, anifrolumab and abatacept, have shown activity in several autoimmune diseases, including SLE, SSc and RA, and are currently in clinical trials. Furthermore, the main aspects of the pathogenesis of SAIDs, including the inhibition of regulatory or cytotoxic T cells and the activation and proliferation of autoreactive B cells, suggest the possibility of therapeutic application of chimeric antigen receptor T (CAR-T) cells to treat autoreactive immune cells.

Some researchers also believe that, in addition to environmental and genetic factors, the gut microbiota is responsible for the pathogenesis of SAIDs [8]. Therefore, in addition to traditional treatments with immunosuppressants and glucocorticoids, as well as more effective approaches associated with the use of biological drugs and CAR-T therapy, there is a view that successful treatment requires measures to correct the intestinal microbiota [9].

At present, CAR-T cell therapy is realizing its potential mainly in oncology, but some progress has also been made in the treatment of SAIDs. According to recent evidence, treating SLE using CD19 CAR-T cells is a viable and highly effective process [10]. Similar data were obtained in patients with severe systemic sclerosis, demonstrating the high potential of this method in clinical practice [11]. Progress in manufacturing CAR-T and other cell immunotherapies has allowed the obtainment of more affordable products for both cancer and autoimmune diseases [12]. Recent developments in CAR-T therapy are anticipated to usher in a new age of autoimmune disease treatment. This article discusses current advances in immunotherapy and future research in this field for the successful treatment of autoimmune diseases, using SLE, SSc and RA as examples.

## 2. Systemic Lupus Erythematosus (SLE)

SLE is an autoimmune disease associated with damage to body tissues and organs due to chronic systemic inflammation. Disruption of immune regulation processes, underlying the pathology, leads to the production of autoantibodies that attack nucleic acids with associated proteins, resulting in damage to the skin, joints, kidneys, blood vessels and other organs [13]. SLE, the most severe form of lupus erythematosus, which can be life-threatening, is accompanied by periods of remission and exacerbations. Another form of lupus is cutaneous lupus erythematosus (CLE), which is divided into several subsets, including chronic cutaneous LE (CCLE) and discoid LE (DLE), the most common type of CCLE with inflamed skin [14,15].

The pathogenesis of SLE remains unclear, and several studies indicate a combination of hereditary, epigenetic and environmental factors. A characteristic feature of SLE is the production of antinuclear antibodies caused by abnormal activation of B and T cells [16]. Since first-line therapies, including glucocorticoids and immunosuppressants, are known to be addictive and cause significant side effects, novel targeted therapies with biological agents may be a good alternative [17]. Biological agents are mainly represented by mAbs. BsAbs are also being developed and used for the treatment of SLE. BsAbs are a subclass of mAbs that can bind two antigens simultaneously. Non-IgG-like BsAbs and IgG-like BsAbs are the two groups of BsAbs that can be separated based on the presence or absence of Fc segments [18,19]. The advantage of BsAbs is that they block multiple activation pathways to disrupt two related or unrelated antigens, providing a broader spectrum of inhibition [20]. In the case of SLE patients, this may lead to a more comprehensive and effective treatment where multiple cytokine pathways contribute to disease activity. The use of bsAbs in clinical practice may be in demand for the management of SLE and other SAIDs, but significant research efforts should be aimed at reducing the side effects of their use [7]. BsAbs can have stronger immunogenicity than simple mAbs because they are mostly fragment-based and not native formats, and the first challenge is to overcome this immunogenicity. Improving the specificity of key targets is also necessary to successfully advance the development of BsAbs [15].

B lymphocytes are characterized by the presence on their membranes of special receptors that recognize foreign proteins—antigens—which contribute to the production of special antibodies against them [21]. Inflammation and tissue damage occur when autoantibodies are produced against the body’s own proteins. In addition to producing autoantibodies, B cells secrete cytokines and promote the activation and differentiation of autoreactive T cells through their antigen-presenting function [22]. Because of these properties, the pathophysiology of SLE is largely determined by the activity of B cells. In this context, the first goal in targeted therapy for SLE is to develop biological agents that block B-cell-related receptors and prevent the activation of cytokines and signaling molecules [15]. The maturation, differentiation, antibody production and autoimmunity of B cells is regulated by the B cell activating factor (BAFF), which belongs to the tumor necrosis factor (TNF) family. This cytokine binds to three receptors (BAFF-R, TACI and BCMA) and prevents B cell deletion [23]. BAFF levels have been correlated with SLE severity [24]. APRIL (a proliferation-inducing ligand), which is homologous to BAFF, promotes BAFF-like activity after forming heterotrimers with BAFF [25,26]. BAFF/APRIL inhibition is one of the targeted pathways in SLE therapy [15]. The first biologic approved by the FDA for SLE is belimumab, a fully humanized mAb that binds to soluble BAFF and prevents it from attaching to the three receptors (Table S1) [27]. In two large Phase III trials, this antibody was shown to reduce the severity of SLE and was generally well tolerated by patients, with no serious or severe adverse events compared to the control group [28,29]. Other agents targeting the BAFF/APRIL family in development and clinical trials are shown in Table S2.

Type I interferon (IFN) (IFNα), produced mainly by plasmacytoid dendritic cells (pDCs), has also been shown to play an important role in the pathogenesis of SLE [30]. IFNα activity is known to be mediated by TLR7 and TLR9 expressed by pDCs [31]. Through this signaling pathway, secreted IFNα activates the transcription of hundreds of different genes involved in autoimmunity [32,33]. In a murine model of lupus, TLR7 and TLR9 were found to participate in the accumulation of antibodies to RNA- and DNA-containing autoantigens [34]. These data suggest that both TLRs and IFNα can be targeted in SLE therapy [35]. Anifrolumab, an anti-IFN-α MoAb, is approved by the FDA for SLE in 2021 and is currently in Phase III clinical trials (Table S1). In recent years, several new drugs targeting TLR7/8 have been developed and are in clinical trials, the most promising of which is the small molecule E6742 [35]. The development of biological therapies targeting TLR7/8 is quite possible in the future.

CD20, a transmembrane calcium channel, and CD22, a receptor on the surface of the B cell membrane, were found to promote B cell proliferation, activation and differentiation [36,37,38]. Blocking the CD20 and CD22 membrane receptors to inhibit B cell proliferation and reduce the inflammatory response is also one of the targets for biological agents [39,40]. Rituximab, an anti-CD20 mAb, is now in Phase IV clinical trial (Table S2). Effectiveness of belimumab after rituximab in SLE was also evaluated in Phase II clinical trial [41].

Another transmembrane protein that promotes the growth, activation, proliferation and signaling of B lymphocytes and acts as a co-receptor for the B cell antigen receptor (BCR) is CD19. When CD19 is linked to BCR, it synergistically increases B cell proliferation, calcium release and mitogen-activated protein kinase activity [42]. There is also a receptor, Fcγ (FcγR) IIB, on the surface of B cells, which has some affinity for IgG and regulates humoral immunity by negative feedback. Together with the Fc region of IgG, it reduces the generation of autoantibodies [43]. Obexelimab, often referred to as XmAb5871, is an anti-CD19 and FcγRIIb BsAb (Table S2). It has been shown to inhibit B cell proliferation, calcium transport and co-stimulatory molecule production in SLE patients. This leads to a reduction in IgM, IgG and IgE levels and suppression of humoral immunity [44]. Results from a Phase II clinical trial showed that in 104 patients treated with obexilimab, B-cells were reduced by approximately 50%, suggesting the efficacy and safety of obexilimab [45].

CXCL13, a small chemokine, is known to promote the migration of B lymphocytes through communication with its receptor, CXCR5, and may be considered part of the pathogenesis of SLE, as increased serum CXCL13 levels have been demonstrated in SLE patients [46]. These data indicate that CXCL13 appears to be a potential therapeutic target, and mAb 5261 has been preliminarily shown to be able to specifically bind and neutralize the activity of CXCL13 [47].

A significant role in the pathogenesis of SLE is played by T cell abnormalities, especially those of T helper cells, also known as CD4+ cells [31]. T-follicular helper (Tfh) was found to support B cell differentiation and activation through the generation of IL-21 in SLE patients and mouse models [48]. T helper 1 (Th1) is predominant in SLE, and the abnormal production of oxidative stress-related inflammatory cytokines, such as IL-12 and IFN-γ, caused by Th1 in SLE patients has also been identified [49,50]. On the other hand, peripheral blood from SLE patients was found to have fewer IL-4-producing Th2 cells, indicating their potential protective function and suggesting that SLE severity may be related to a higher IFNγ/IL-4 ratio [51]. T helper 17 (Th17) cells, the main source of IL-17, are also involved in the pathogenesis of SLE. These cells have been shown to promote activation of the innate immune system, neutrophil recruitment and B lymphocyte function, and their proinflammatory properties can worsen tissue damage [52]. Additionally, SLEDAI scores in patients with lupus nephritis were shown to correlate with IL-17 levels [53,54]. Immune tolerance was also influenced by CD4+ Treg cell abnormalities in SLE patients, where an increased rate of Treg apoptosis was correlated with disease severity [55]. The IL-2/STAT5 signaling pathway and IL-2 level was shown to regulate the number and function of CD4+ Treg cells [56]. In mice and SLE patients, treatment with low-dose recombinant IL-2 was found to promote Treg cell-mediated beneficial activities in disease manifestations and to raise the circulating Tfr/Tfh ratio, which was associated with increased renal damage and reduced anti-dsDNA titers. Recovery of the Tfr/Tfh immune balance in SLE patients by IL-2 therapy was confirmed by a special study [57,58].

Other pro- and anti-inflammatory cytokines, produced by B and T lymphocytes, such as IL-6, IL-10, IL-12 and IL-18, are also involved in SLE pathogenesis, and elevated levels of these cytokines in the serum of SLE patients were found to correlate with the disease activity [59,60,61]. Targeting of cytokines is one more approach in addition to the above-mentioned SLE immunotherapies. Novel biological agents that inhibit cytokines, such as IL-6, IL-17, IFNɑ, TNFɑ and IL21/IL23, have been developed in the last few years and are objects of clinical trials (Table S2).

Despite significant advances in the treatment of SLE with antibodies, a Phase II/III SLE trial that evaluated rituximab showed a subgroup of patients (9.5%) that did not achieve B cell depletion [62]. Also, the survival of B cells in lymph nodes after rituximab treatment was observed [63]. Additionally, plasmablasts and long-lived plasma cells implicated in the generation of autoantibodies in SLE cannot be depleted by rituximab because they do not express CD20 [64]. These results suggest limitations of SLE therapy with rituximab in certain severe forms of the disease. In this respect, SLE therapy to treat CD19+ B lymphocytes may be quite a beneficial approach and using anti-CD19 CAR-T cells would be a proper decision. Genetically engineered redirected T lymphocytes are known as CAR-T cells and to bind and recognize particular antigens on target cells for which they have a specialized membrane receptor [65]. To make CAR-T cells, CAR genes are introduced into the genome of T cells after they have been isolated from a patient’s peripheral blood. Once produced, the CAR-T cells are grown and reinfused into the patient [66]. Because they may reach deep tissues, anti-CD19 CAR-T cells provide an additional benefit over anti-CD20 mAb treatment.

**Figure 1:**
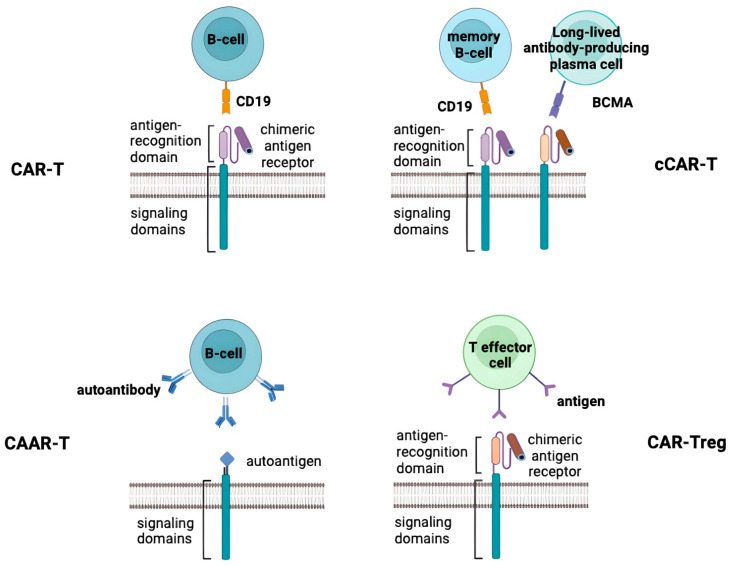
Four different approaches in CAR-T cell therapy for treatment of SAIDs. Top-left panel shows a CAR-T which recognizes CD19 on the target B cell and induces a cytotoxic effect. Top-right panel shows a compound CAR-T (cCAR-T), which is a two-unit CAR consisting of a complete BCMA-CAR fused to a complete CD19-CAR, allowing independent expression of both CAR receptors separately on the T cell surface to target two cell types: CD19+ memory B cells and BCMA+ plasma cells. Bottom-left panel shows a CAAR-T cell that recognizes an autoantibody on the surface of the target B cell and induces a cytotoxic effect. Bottom-right panel shows a CAR-Treg that recognizes an antigen on the target cell and induces a regulatory response.

Targeting B cells and their malignant offspring using their highly specific and prevalent surface antigen CD19 is currently the most advanced method of CAR-T cell therapy, notably in the treatment of cancer (Figure 1) [65,67]. According to preclinical studies, anti-CD19 CAR-T cells have shown efficacy in the treatment of SLE, initially in a mouse model. According to these studies, treatment of SLE with anti-CD19 CAR-T cells reduce the number of B cells, stops the formation of autoantibodies and reverses organ symptoms [68,69]. Also, the rapid disappearance of dsDNA autoantibodies and clinical remission were achieved in a single patient with severe and refractory SLE exacerbated by active nephritis, suggesting the principal feasibility of this type of therapy for the treat of SLE and other autoimmune diseases [70].

Based on these studies, five SLE patients were recruited into a special CAR-T cell project [10]. This study revealed a significant reduction in B-cell counts, normalization of clinical parameters and improvement in laboratory measures, including a reduction in anti-dsDNA antibodies. After three months, all five patients had achieved stable remission of SLE according to the DORIS criteria. This study indicates that SLE therapy with anti-CD19 CAR-T cell is a tolerable, feasible and quite effective approach; however, there is a need for larger-scale placebo control trials [71]. Two clinical trials are currently registered to evaluate the safety and efficacy of anti-CD19 CAR-T cells in the treatment of SLE patients: NCT03030976, using 4-1BB (CD137) as a co-stimulator, and NCT05765006. Both are in Phase I.

In another study, a patient with a 20-year history of SLE complicated by stage IV diffuse large B-cell lymphoma was treated with compound CAR-T (cCAR-T) co-expressing anti-BCMA and anti-CD19, targeting both CD19+ memory B cells and BCMA+ plasma cells (Figure 1). Long after treatment, plasma cell depletion and stable remission were still observed, and anti-nuclear antibody titers were undetectable [72]. There are currently two Phase I clinical trials evaluating the safety and efficacy of CD19/BCMA cCAR-T cells in the treatment of refractory SLE: NCT05030779 and NCT05474885.

Two novel therapeutic variants of CAR-T cells have been proposed for autoimmune diseases that may be useful in the treatment of SLE. One of them is chimeric autoantibody receptor T cells (CAAR-T cells) which have a high affinity for an autoantibody on the B cell surface and generate a cytotoxic effect (Figure 1). And another one is chimeric antigen receptors in regulatory T lymphocytes (CAR-Tregs) that bind to a specific antigen in a target cell to execute the activation and regulatory function of Tregs (Figure 1) [73]. Targeting autoreactive B cells, CAAR-T comprises a particular antigen and an intracellular signaling domain. It is a variant of modified CAR (Figure 1). Unlike the scFv domain, the CAAR determines the selective cytotoxicity of CAAR-T cells solely against immune cells that carry receptors to certain autoantigens without overall immunosuppression. The therapeutic effect of CAAR-T cells is that they bind to specific autoantibodies expressed on the surface of B cells and kill cells [74]. For the first time, CAAR T cells recognizing the pemphigus vulgaris (PV)-specific autoantigen, desmoglein 3, were successfully tested to eliminate autoreactive B cells in a mouse model of PV. Successful target cell lysis has also been observed in the treatment of autoimmune encephalitis patients using CAAR-T cell therapy [75]. However, there are no data on the use of this cell type for the treatment of lupus.

Since Tregs are usually suppressed in SAIDs, another effectual approach for the restoration of immune tolerance implies “switching” T cell phenotypes from cytotoxic to regulatory. Without causing systemic immunosuppression, CARs drive Tregs to the location of autoimmune activity, enhancing their suppressive capacity [76]. CAR-Tregs are CAR-T cells that have been transformed into Tregs by the transduction of FOXP3, which regulates pathways involved in the formation and operation of regulatory T cells, coupled with CAR [77]. CAR-T cell proliferation and persistence may be enhanced by FOXP1 deletion, while autoimmune abnormalities caused by aberrant B-cell responses may be reduced by lowering FOXP1 levels [78]. Through clonal deletion, immunological ignorance and anergy induction, CAR-Tregs are thought to identify and control autoimmune T cells [79]. In addition, CAR-Tregs were found to produce immunomodulatory cytokines, such as TGF-β, IL-10 and IL-35, and to promote apoptosis of Teff cells via granzyme B/A, Fas ligand perforin, thus preventing the activity of Teff cells [80]. Strong proinflammatory conditions can transform Treg cells into cells that produce IL17 or other inflammatory cytokines, and, as has been suggested, CAR-Treg cells may be effective in the treatment of SLE under the condition of eliminating the influence of inflammatory cytokines, such as IL6, IL21 and IFNɑ, which inhibit Treg activity [81].

## 3. Systemic Sclerosis (SSc)

SSc has a similar pathogenesis to SLE, with the differences being specifically characterized by endothelial cell injury, connective tissue deposition, fibrosis and vasculopathy [82]. In addition to suppressing the immune system with general immunosuppressants, immunotherapy for SSc has recently also included targeted therapy aimed at blocking specific molecules involved in the activation of B and T lymphocytes.

B cells were found to play a significant pathogenic role in SSc, including autoantibody production, cytokine synthesis and antigen presentation. MAbs and BsAbs, as B cell activity inhibitors, are currently in clinical trials for the treatment of SSc. CD19, a key regulator of B cell signaling, was found to be overexpressed in both memory and naive B cells in SSc and therefore associated with their hyperreactivity [83]. In a mouse model of SSc, CD19 suppression was shown to reduce skin thickness, collagen production and autoantibody levels [83]. Inebilizumab, an anti-CD19 mAb, is currently in a Phase III clinical trial (Table S2). Similar to SLE, B cell targeting with the anti-BAFF mAb belimumab is another approach in SSc therapy (Table S2). Belimumab was shown in a Phase II clinical trial to reduce median modified Rodnan skin thickness score (MRSS) and expression of profibrotic genes [84].

As a high level of soluble CD30 was found to correlate with disease activity in patients with diffuse cutaneous SSc, CD30 is also considered a marker of activated lymphocytes and can be targeted [85]. Brentuximab vedotin anti-CD30 mAb is currently in Phase II clinical trials (Table S2).

In addition to B cells, the role of T cells in inflammation and fibrosis in SSc has also been discussed, and their activation in SSc is evidenced by the accumulation of T cell cytokines IL-4 and IL-13 and the early presence of IgG autoAbs, as well as T cell infiltrates in the skin prior to fibrosis [86]. Romilkimab BsAb, which targets both IL-4 and IL-13, is now in Phase II trials (Table S2).

The involvement of T cell mediated cytokines, such as IL-1, IL-4, IL-13, IL-17, IL-23 and IL-31, in the regulation of inflammation and in vascular and fibrotic changes has led to their being targeted with biological agents [87]. IL-6 is considered an important target because its elevated levels have been associated with poor prognosis in SSc [88].

Another potential target for the treatment of SSc is transforming growth factor-β (TGFβ), which consists of three isoforms and has been shown to have profibrotic activity and promote collagen synthesis [89]. Fresolimumab, an anti-TGFβ mAb, is in Phase I clinical development (Table S2).

IL-17 has also been shown to contribute to the vasculopathy, inflammation and fibrosis that occurs in SSc [90]. Brodalumab anti-IL-17 mAb shows efficacy in SSc and reduces MRSS in patients, and Phase III trials have been initiated (Table S2).

Serum and skin tissue from patients with SSc were shown to have elevated levels of both isoforms of IL-1, which can stimulate fibroblast proliferation and collagen production [91]. Bermekimab, an anti-IL-1a mAb, demonstrated some efficacy in a Phase II study, suggesting its potential to target the disease (Table S2).

As blocking IL-31/IL-31RA has been shown to reduce cytokine release and fibrosis in SSc patients and IL-31/IL-31RA is overexpressed in patient dermal fibroblasts, this mediator may be a therapeutic target [92]. The anti-IL-31 mAb nemolizumab is now entering Phase II clinical trials (Table S2).

Blocking the IFN signaling pathway was suggested to reduce fibrosis and inflammation in SSc and may therefore be a target [93]. Similar to SLE, the anti-IFN-α mAb anifrolumab has shown efficacy in the treatment of SSc and is now in a Phase III clinical trial (Table S2).

IL-23 level has also been shown to be elevated and correlate with lung fibrosis in patients and animal models [94,95]. The anti-IL-23 mAb guselkumab is currently in a Phase II trial in patients with SSc (Table S2). As in instances of SLE, CD20+ B cell depletion was speculated to be insufficient in SSc; moreover, plasmablasts, which may also be responsible for autoantibody production, are targeted via CD19 [96]. Until now, CAR-T cell therapy has been used almost exclusively for cancer. However, the recent study in SLE is not only a potential game changer for the treatment of SLE with anti-CD19-targeted CAR-T cells, but it is also very pertinent to SSc [10].

The first anti-CD19 CAR-T cell treatment was reported in a patient with severe refractory SSc with fibrosis of the skin, lung and heart [96]. This patient demonstrated significant improvement in carpal arthritis three months after starting treatment. Skin, heart and joint fibrosis also showed a tendency of improvement, and less frequent and less severe attacks of Raynaud’s phenomenon were reported. These findings indicate that B cell-mediated autoimmunity plays a significant role in severe SSc and support the early idea that CD19-targeting CAR-T cell therapy may be useful in treating SAIDs. Clinical trials for the use of CAR-T therapy in the treatment of SSc are expected to follow soon. Currently, a Phase I clinical trial (NCT05085444) has begun to evaluate the safety and efficacy of anti-CD19/BCMA CAR-T in the treatment of patients with refractory scleroderma.

## 4. Rheumatoid Arthritis (RA)

Rheumatoid arthritis (RA) is a chronic systemic inflammatory disease of an autoimmune nature with a variable clinical course, characterized by progressive destruction of the synovial joints, accompanied by cartilage and bone destruction and disability. Although treatment for RA has long consisted of traditional anti-rheumatic drugs, such as glucocorticoids and nonsteroidal anti-inflammatory drugs, targeted synthetic and biological drugs are now available. The main focus of our review is on biological agents. The use of biological agents has now fundamentally changed the clinical options for RA therapy. The main targets of these agents are inhibitors of B- and T-lymphocyte activity and cytokines, especially tumor necrosis factor (TNF)-α and IL-6 [97,98,99,100].

Along with other autoimmune diseases, the main B cell depletion strategy is anti-CD20 antibody therapy [101]. Rituximab has demonstrated efficacy in patients with RA [102]. This antibody was approved in 2006 for the treatment of RA (Table S1).

IL-6 stimulates neutrophil recruitment to the joints during the early stages of RA, which facilitates the following infiltration of monocytes into the synovial fluid [103]. Excessive and persistent joint inflammation damages tissue via bone erosion, activation of osteoclasts and cartilage damage. Additionally, IL-6 directly promotes osteoclast activity by causing synovial cells to produce RANKL [104]. Anti-IL-6 mAb tocilizumab was registered for the treatment of RA in the United States in 2010 (Table S1).

Sarilumab is another anti-IL-6R mAb with improved activity compared to tocilizumab and was approved for RA in the US, EU and Japan in 2017 (Table S1) [105].

Infliximab is the first anti-TNF-α mAb that was approved for RA therapy by the FDA in 1998 (Table S1) [106]. Other anti-TNF-α agents already approved for the treatment of RA include adalimumab, certolizumab pegol, etanercept and golimumab (Table S1).

IL-1β levels in the plasma and synovial fluid of RA patients were found to be strongly associated with disease severity [107]. Anakinra, which is an IL-1 inhibitor, a modified version of the human interleukin-1 receptor antagonist protein, has been shown to be a relatively safe and modestly effective biological therapy for RA [108]. This drug was approved in 2001 for the treatment of RA (Table S1).

Similar to SLE, an increased CXCL13 serum concentration in RA patients was demonstrated and therefore it can be regarded as part of the pathogenesis of RA and a promising target for therapy [46].

Other biological agents that are inhibitors of B- and T-lymphocyte activity and cytokines currently in development and clinical trials are listed in Table S2.

Recently, anti-CD19 CAR-T cells were successfully utilized in treating patients with SLE and SSc [10,96]. A special CAR-based approach has been proposed to treat RA and has shown good results when tested in vitro [109].

Since a CAR or CAAR only targets a single cell type, its applicability to RA, which often exhibits a variety of autoreactive responses, is limited. One idea was to develop a customized therapeutic strategy using a universal CAR-T cell system that allows targeting of various types of autoreactive B cell subsets by T cells expressing a single scFv combined with known autoantigen peptides according to patients’ specific autoantigen profiles [109]. The authors used an approach previously developed for cancer therapy [110]. Anticitrullinated protein antibodies (ACPAs) are some of the most specific serological markers for RA that are linked to the onset of the illness [111]. Four autoantibody-positive citrullinated peptides, including citrullinated vimentin, citrullinated type II collagen, citrullinated fibrinogen and tenascin C, were selected as mediators for targeting autoreactive B cells by redirection of CAR-Ts. For this purpose, mediators were conjugated with fluorescein isothiocyanate (FITC) and anti-FITC CAR-Ts were prepared as described in an earlier study [112]. In vitro results showed that the engineered T cells effectively targeted and eliminated autoreactive B cells expressing autoantibodies to citrullinated vimentin, citrullinated type II collagen, citrullinated fibrinogen and tenascin C [109]. The specificity of the T cells was confirmed by their recognition of the FITC-labeled autoantigenic peptide epitopes. This approach holds promise for the development of targeted therapies for autoimmune diseases characterized by the presence of autoreactive B cells. By selectively eliminating these pathogenic B cells, it may be possible to mitigate the immune response and reduce disease severity. Further studies are warranted to optimize this strategy and evaluate its efficacy in preclinical models and eventually in clinical trials. Overall, not only RA but other SAIDs may benefit from this innovative approach.

## 5. Common Therapeutics for SLE, SSc and RA

**Figure 2:**
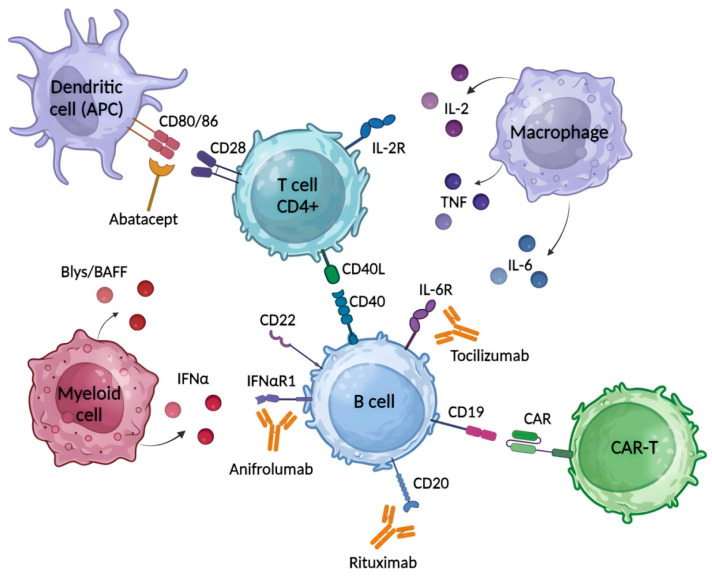
The basic common targets for the treatment of SLE, SSc and RA with biological agents. Rituximab is chimeric anti-CD20 mAb, which eliminates autoreactive B cells and a subset of T cells. Abatacept is a fusion protein, a soluble CTLA-4 analog, that inhibits T cell activation by binding to CD80 and CD86 receptors on APC, selectively blocking the specific interaction of CD80/CD86 receptors to CD28 and, therefore, inhibiting T cell proliferation and B cell immunological response. Anifrolumab is an immunoglobulin gamma 1 kappa (IgG1κ) mAb that selectively binds to subunit 1 of INFAR1, inhibiting receptor activity and reducing downstream signaling and gene transcription of inflammatory mediators. Tocilizumab is an anti-IL-6R mAb that binds soluble and membrane-bound IL-6 receptors, preventing IL-6 mediated inflammation. The use of CAR-T cells targeting CD19 is the most promising approach for treatment of SLE, SSc and RA.

Some biological agents, such as rituximab, abatacept, anifrolumab and tocilizumab, have been shown to be effective in several SAIDs, including SLE, SSc and RA, discussed above, and are currently in clinical trials (Figure 2). The first on this list is rituximab (Table S3). It is a chimeric anti-CD20 mAb that eliminates autoreactive B cells and a subset of T cells, thereby reducing antibody production [113]. Rituximab has demonstrated efficacy in patients with RA and was approved by the FDA in 2006 for the treatment of RA [102]. However, its efficacy in SLE has not been clearly demonstrated, and future studies are needed to clarify its efficacy in this disease [62,63]. Rituximab showed clear promise in SSc in the first randomized controlled trials, acting on the skin as well as the lungs [114,115,116]. However, the long-term efficacy of this drug in the treatment of patients with SSc is still controversial and needs to be clarified [117]. Clinical trials of the drug have already reached Phase IV in SLE and Phase III in SSc (Table S3). Adding belimumab after rituximab in a special trial of SLE therapy significantly reduced the risk of severe flares and serum IgG anti-dsDNA antibody levels, suggesting that combining these drugs could be a promising therapeutic strategy [41]. The combination of belimumab and rituximab is also being studied in a Phase II trial in SSc patients (NCT03844061).

B cell activation is regulated by the interaction of co-stimulatory signals with T cells when CD28, which is constitutively expressed on T cells, binds to CD80/CD86 on antigen presenting cells (APCs). At the same time, cytotoxic T lymphocyte-associated protein 4 (CTLA-4) was found to compete with CD28 for CD80/CD86 binding, and an increase in CTLA-4 expression on the activated T cell provides negative signals for T cell activation [118]. This approach is currently being used to target SAIDs, including SLE, SSc and RA (Figure 2). For example, the fusion protein abatacept, a soluble CTLA-4 analogue, acts as an antagonist of CD28-mediated co-stimulation to prevent the specific interaction of CD28 with CD80/CD86, resulting in inhibition of B-cell growth and activation (Table S3). Abatacept has been shown in long-term studies to be a well-tolerated drug with an acceptable and consistent safety profile for the treatment of RA. Immunogenicity rates are low and transient and do not interfere with clinical response or safety [119]. And so, the FDA approved it for RA in 2005. Abatacept has demonstrated efficacy and an acceptable safety profile in the treatment of SLE patients [120]. This drug is also being studied for the therapy of SSc and is currently in clinical trials (Phase II), in which it has showed some improvement in skin fibrosis and MRSS score [121,122].

Type 1 INF (IFNα) has also been shown to play an important role in the pathogenesis of SAIDs and is of increasing interest to researchers as a therapeutic target (Figure 2) [30,123,124]. Anifrolumab is an immunoglobulin gamma 1 kappa (IgG1κ) mAb that targets subunit 1 of INFAR1 and therefore blocks IFN-α and IFN-β signaling (Table S3) [124]. A meta-analysis of four study databases evaluating the safety of anifrolumab versus placebo in SLE patients clearly demonstrated the tolerability and efficacy of this antibody [125]. As a result, anifrolumab was approved for clinical use first in the United States in July 2021 and then in the European Union in February 2022 for the treatment of SLE. Anifrolumab is currently in Phase III clinical trials for this disease (Table S3). In SSc, blocking the IFN signaling pathway has been suggested to reduce fibrosis and inflammation and may therefore be a target [93]. Similar to SLE, anifrolumab has shown efficacy in the treatment of SSc and is now in a Phase III clinical trial (Table S3). Clinical trials for RA reported that future larger studies are needed to assess the efficacy of this drug in RA [123,126].

Tocilizumab is an anti-IL-6R mAb that binds soluble and membrane-bound IL-6 receptors and prevents IL-6-mediated inflammation (Figure 2). This antibody has shown some efficacy in patients with refractory RA [127]. In addition, tocilizumab monotherapy has been shown to be more effective in the treatment of RA than treatment with adalimumab, a TNF-α antagonist [128]. Based on its efficacy, tocilizumab was approved for the treatment of RA in Japan in 2008, in the European Union in 2009 and in the United States in 2010 (Table S3). The efficacy of tocilizumab in SSc was confirmed in Phase III clinical trials [129,130]. Tocilizumab is currently in Phase I clinical trials for SLE (Table S3). Preliminary data have shown that tocilizumab may be a good alternative treatment for patients with SLE who do not respond to high-dose glucocorticoids, but further studies are needed [131,132].

CAR-T therapy is the most advanced and promising approach for the treatment of SLE, SSc and RA, and after appropriate research and refinement, any of the described cell types (anti-CD19 CAR-T, cCAR-T, CAAR-T and CAR-Treg) may be applicable (Figure 1 and Figure 2).

## 6. Conclusions and Perspective

Despite significant advances in the management of SAIDs, some patients are at high risk of serious complications and side effects from treatment because they do not respond to the current standard of care. As a result, new alternatives have emerged, such as immunotherapy. SAIDs, such as SLE, SSc and RA, share common molecular targets for antibody treatment, including B cells, T cell or T cell co-stimulator blockers, and pro-inflammatory cytokines or cytokine receptors. Some biological agents, such as rituximab, tocilizumab, anifrolumab and abatacept, have been shown to be effective in several SAIDs, including SLE, SSc and RA, discussed above, and are currently in clinical trials. But even therapeutics using biological agents have been found to be not entirely efficacious. The generation of CAR-T cells offers a novel means to overcome some of the drawbacks of antibody therapy, such as immunogenicity, side effects from repeated injections and incomplete autoantibody clearance. Recently, anti-CD19 CAR-T cells were successfully utilized in treating patients with SLE and SSc, clearing the way for treating RA and other SAIDs. Advances with this therapy have shown very encouraging results. This approach holds great potential for improving the lives of patients with autoimmune diseases. In tissues affected by RA and other SAIDs, CAR-Treg cells may be a viable treatment to restore immune tolerance. CAR-T cells can also be used to precisely deplete a number of autoreactive B cells. Furthermore, the use of different targets for these modified T cells allows for fine-tuning of their function, providing a personalized approach to treatment. This innovative approach holds great promise for improving outcomes for patients affected by SAIDs. Further preclinical approaches and clinical studies are needed to fully explore the potential of cCAR-T, CAR-Treg and CAAR-T cell therapy, but the initial results are highly encouraging.

The choice of treatment is case-specific. It depends on the severity of the disease and other associated conditions. By understanding the unique characteristics of each therapeutic agent, healthcare professionals can optimize therapy outcomes and alleviate symptoms more effectively. Despite the obvious advantages and successes associated with the use of biologics and CAR-T cells for the treatment of autoimmune diseases, both methods have their drawbacks. Biologics offer more selective outcomes with fewer toxic effects than traditional treatments with glucocorticoids and immunosuppressants [126]. However, despite the significance of currently available therapies, achieving the effective and permanent restoration of immune homeostasis is still challenging considering that repeated antibody injection is generally needed, and an insufficient antibody dose leads to incomplete depletion and treatment failure. In addition, the immunogenicity caused by long-term administration of antibodies remains a concern [133]. Immunogenicity limits the use of biological drugs. It has been found that repeated injection of recombinant homologues of some human proteins, such as IFNs or erythropoietin, especially when aggregated or partially denatured, leads to the production of an anti-drug antibody (ADA) response [126]. ADAs can adversely affect the pharmacokinetics, bioavailability and efficacy of biologics and in some cases may neutralize their activity. ADAs can also cause immune complex disease, allergic reactions and, in some cases, severe autoimmune reactions [133]. Assessment of immunogenicity is therefore an important component of drug safety evaluation. Concomitant therapy with immunosuppressive drugs can also influence a patient’s immune response to a biopharmaceutical. The duration of treatment and the route of administration also influence the immune response to a biological drug. CAR-T cell therapy has serious side effects when used in cancer patients, causing the potentially fatal cytokine release syndrome (CRS). Because the immune system must be depleted before CAR-T cells are administered, patients are also at risk of infection. However, the risk of CRS in SAIDs may be reduced by specific therapies that reduce the number of circulating B cells, such as the biological agents described above [134].

Therapeutic mAb- and BsAb-based therapy is more flexible and versatile than CAR-T cell-based immunotherapy, since Abs were not developed to be personalized treatments for patients and therefore are more accessible at a much lower cost [135]. Antibody therapy also allows easier dosage control and adjusted treatment regimens depending on the patient’s response [136]. Also, compared to CAR-T cell-based immunotherapies, antibodies appear much more widely applicable owing to the simplicity of application, the reproducibility of results and scalability for mass production [137]. CAR-T cell therapy requires a complex and time-consuming manufacturing process which significantly limits its broad availability, whereas Abs, if approved, are expected to be much more affordable. Another complication of CAR-T cell therapy is the requirement for lymphodepletion prior to the infusion [138]. CAR-T therapies and lymphodepletion are resource-intensive therapies, and more funding, staffing, expertise and hospital beds and higher manufacturing capacity are needed to evaluate all the opportunities [139]. On the other hand, CAR-T therapy is a safer and more effective method of treatment, and CAR-T therapy seems to hold the greatest promise for future successful practice and the curing of patients. Thus, improving the safety and reducing the cost of CAR-T therapy, as well as improving the specificity, safety and reducing the immunogenicity of antibodies and other biological agents, are important goals for future research.

The development of new targeted therapies with different modes of action and favorable side effect profiles that are effective in SAIDs is expected in the near future. The future of immunotherapy in precision medicine is indeed promising, offering hope for improved management of SAIDs and enhanced patient outcomes.

## Supplementary Materials

The following supporting information can be downloaded at: https://www.mdpi.com/article/10.3390/antib13010010/s1. Supplementary Table S1: List of biologics already approved by the FDA for the treatment of SLE, SSc and RA; Table S2: Biologic drugs for treating SLE, SSc and RA that are currently under clinical trials; Table S3: Common biologic agents used to treat SLE, SSc, and RA.

## List of Abbreviations

ADCC	Antibody-dependent cell-mediated cytotoxicity
ACPA	Anticitrullinated protein antibody
APRIL	A proliferating inducing ligand
BAFF	B cell activating factor
BsAb	Bispecific antibody
CAR-T cell	Chimeric antigen receptor T cell
CAAR-T cell	Chimeric autoantibody receptor T cell
CCLE	Chronic cutaneous lupus erythematosus
CDC	Complement-dependent cytotoxicity
CLE	Cutaneous lupus erythematosus
CTLA-4	Cytotoxic T lymphocyte-associated protein 4
DLE	Discoid lupus erythematosus
FDA US	Food and Drug Administration
FITC	Fluorescein isothiocyanate
FLS	Fibroblast-like synoviocytes
GM-CSF	Granulocyte-macrophage colony-stimulating factor
JAK	Janus kinase family
mAb	Monoclonal antibody
SAIDs	Systemic autoimmune diseases
SLE	Systemic lupus erythematosus
TNF	Tumor necrosis factor
RA	Rheumatoid arthritis
