# Supplementary material for: Immunotherapy Strategy for Systemic Autoimmune Diseases: Betting on CAR-T Cells and Antibodies
Source: Antibodies (Basel). 2024 Feb 1;13(1):10. doi: 10.3390/antib13010010 (PMC10885098; doi:10.3390/antib13010010)
Supplement: Supplementary file 1 [file antibodies-13-00010-s001.zip › antibodies-2748422-supplementary.pdf]

Table S1. List of biologics already approved by the FDA for the treatment of SLE, SSc and RA.

Agent	Action cells	Disease	Date of FDA approval	Clinical trials code / Reference number
Belimumab	Anti-BAFF, IgG1 moAb that specifically binds to the soluble form of human B-cell-stimulating protein	SLE	2011	NCT04956484<br>NCT01597492<br>NCT01632241
Anifrolumab	MoAb, Anti-IFN-α receptor	SLE	2021	NCT02794285<br>NCT02446899<br>NCT02446912
Rituximab	Chimeric anti-CD20 moAb. Eliminates B cells, and a subset of T cells	RA	2006	NCT02731560<br>NCT05116228
Abatacept	Protein, CTLA4-Ig construct. CD80/86 costimulation inhibitor, selectively modulates a key co-stimulatory signal for the full activation of T lymphocytes expressing CD28. Inhibits activated Tcells, and antigen-presenting cells.	RA	2005	NCT03882008<br>NCT01758198<br>NCT03492658
Tocilizumab	moAb against IL-6R. Inhibits B cell differentiation, M2 macrophages, TH17 polarization, and myofibroblast activation	RA	2010	NCT02659150<br>NCT02010216<br>NCT01120366
Sarilumab	human anti-IL-6R moAb	RA	2017	NCT03449758
Anakinra	IL-1 inhibitor, it’s a recombinant and slightly modified version of the human interleukin 1 receptor antagonist protein	RA	2001	NCT02236481

Adalimumab	moAb, works by inactivating tumor necrosis factor-alpha (TNFα)	RA	2002	NCT01270087<br>NCT00234897
Infliximab	chimeric human-mouse anti-(TNFα) moAb	RA	1998	NCT02770794<br>NCT00246064
Certolizumab pegol	Fragment of moAb, anti-(TNFα)	RA	2009	NCT00580840
Etanercept	It's a fusion protein produced by recombinant DNA, TNF inhibitor	RA	1998	NCT00913458<br>NCT00768053<br>NCT00099554<br>NCT01783015
Golimumab	Anti-TNFα human moAb	RA	2009	NCT01295151<br>NCT03100253

Table S2. Biologic drugs for treating SLE, SSc and RA that are currently under clinical trials

Agent	Action, Cells	Disease	Phase of trial	Clinical trials code
**B-cell activity inhibitors**				
Blisibimod	Anti-BAFF fusion protein consisting of four BAFF binding domains	SLE	III	NCT01395745<br>NCT02514967
Obexelimab (XmAb5871)	BSAb, targeting CD19 and FcγRIIb on B cells	SLE	II	NCT02725515
Epratuzumab	Anti-CD22 human moAb	SLE	III	NCT01262365<br>NCT01408576

				NCT00383214<br>NCT00111306
Obinutuzumab	Human anti CD20 moAb	SLE	III	NCT04963296
Mosunetuzumab	BsAb, binds CD20 and CD3 to engage T-cells	SLE	I	NCT05155345
PRV-3279	Fv specific BsAb, simultaneously ligate the inhibitory molecule CD32B (FcγRIIb) with the B-cell receptor component CD79B on B cells	SLE	II	NCT05087628
Telitacicept	Fully human TACI-Fc fusion protein that targets B lymphocyte stimulator (BLyS) and a proliferating-inducing ligand (APRIL)	SLE	IV	NCT05899907
Ocrelizumab	Anti CD20 moAb	SLE	III	NCT00539838<br>NCT00626197
Abetimus	It is made of four double-stranded oligodeoxyribonucleotides that are attached to a carrier platform and are designed to block specific B-cell anti double stranded DNA antibodies	SLE	III	NCT00089804<br>NCT00035308
Rozibafusp Alfa (AMG-570)	BsAb, targeting inducible costimulator ligand (ICOSL) and B cell activating factor (BAFF)	SLE	II	NCT04058028
		RA	I	NCT03156023
Ianalumab	MoAb, anti-BAFFR	SLE	III	NCT05639114<br>NCT05624749
		RA	I	NCT03574545
Atacicept	Recombinant fusion protein, anti-BLyS and APRIL	SLE	III	NCT00624338
		RA	II	NCT00430495<br>NCT00595413
Tabalumab	Anti-BAFF moAb	SLE	III	NCT02041091

		RA	III	NCT01676701<br>NCT01202760
Rituximab	Chimeric anti-CD20 moAb. Eliminates B cells, and a subset of T cells	SLE	IV	NCT05828147
		SSc	III	NCT01748084<br>NCT04274257
Brentuximab vedotin	Anti-CD30 moAb	SLE	II	NCT02533570
		SSc	III	NCT03222492<br>NCT03198689
Inebilizumab	anti-CD19 monoclonal antibody	SSc	III	NCT05198557
Belimumab	Anti-BAFF, IgG1 moAb that specifically binds to the soluble form of human B-cell-stimulating protein	SSc	II/III	NCT05878717<br>NCT0384406<br>NCT01670565
**T-cell or T-cell co-stimulator blockers**				
Itolizumab	Anti-CD6 moAb	SLE	I	NCT04128579
Iscalimab	Anti-CD40 moAb	SLE	II	NCT03656562
Milatuzumab	Anti-CD74 moAb	SLE	I	NCT01845740
Lulizumab pegol	Anti-CD28 moAb	SLE	II	NCT02265744
Acazicolcept	Anti-CD28 and ICOS BsAb	SLE	II	NCT04835441
LY3361237	Agonist moAb to the checkpoint inhibitory receptor BTLA (B and T lymphocyte attenuator)	SLE	II	NCT03933943
Dapirolizumab pegol	Anti-CD40L moAb	SLE	III	NCT04294667
Dazodalibep (VIB4920)	anti-CD40 ligand-Tn3 fusion protein	SLE	II	NCT02804763
		RA	II	NCT04163991<br>NCT02780388<br>NCT04129164

BI655064	Anti-CD40 moAb	SLE	II	NCT03385564<br>NCT02770170
		RA	I	NCT01751776
Abatacept	Protein, CTLA4-Ig construct. CD80/86 costimulation inhibitor, selectively modulates a key co-stimulatory signal for the full activation of T lymphocytes expressing CD28. Inhibits activated Tcells, and antigen-presenting cells.	SLE	III	NCT00430677
		SSc	II	NCT00442611<br>NCT02161406
Romilkimab	BsAb against IL-4/IL-13 cytokines, which are derived from Th2 cells, mast cells eosinophils, innate lymphoid cells. Inhibits M2 macrophages, and myofibroblasts.	SSc	II	NCT02921971
Theralizumab (TAB08)	anti-CD28 moAb	RA	II	NCT01990157
**Targeting cytokine or cytokine receptor**				
Sifalimumab	Anti-IFN-α moAb	SLE	II	NCT00979654<br>NCT01283139
Rontalizumab	Anti-IFN-α moAb	SLE	II	NCT00962832
JNJ-55920839	Anti-IFN-α moAb	SLE	I	NCT02609789
IFN-α kinoid	A therapeutic vaccine composed of IFNα2b coupled to a carrier protein	SLE	II	NCT02665364
BT-063	Anti-IL-10 moAb	SLE	II	NCT02554019
Vunakizumab (SHR1314)	Anti-IL17A moAb	SLE	II	NCT04924296
Avizakimab (BOS161721)	Anti-IL21 moAb	SLE	II	NCT03371251
Infliximab	Chimeric human-mouse anti-(TNFα) moAb	SLE	II/III	NCT00368264

Etanercept	It's a fusion protein produced by recombinant DNA, TNF inhibitor	SLE	II	NCT02656082
Ustekinumab	MoAb that binds to the p-40 subunit of both IL-12 and IL-23 so that they subsequently cannot bind to their receptors to activate T-cells	SLE	III	NCT03517722
		RA	II	NCT01645280
Secukinumab	anti-IL17A moAb	SLE	III	NCT05232864<br>NCT04181762
		RA	III	NCT01770379<br>NCT01377012<br>NCT01350804
ALX-0061	anti-IL-6R BsAb	SLE	II	NCT02437890
		RA	II	NCT02518620<br>NCT01284569
Guselkumab	anti-IL23 moAb	SLE	II	NCT04376827
		SSc	II	NCT01645280
Tocilizumab	moAb against IL-6R. Inhibits B cell differentiation, M2 macrophages, TH17 polarization, and myofibroblast activation	SLE	I	NCT05835986
		SSc	III	NCT01532869<br>NCT02453256<br>NCT05963048
Brodalumab (KHK4827)	anti-IL17A moAb	SSc	III	NCT03957681
Bermekimab	anti-IL-1a mAb	SSc	II	NCT04045743
Nemolizumab	anti-IL-31 mAb	SSc	II	NCT05214794
Fresolimumab	anti-TGFβ mAb	SSc	I	NCT01284322

Sirukumab	anti-IL-6 moAb	RA	III	NCT01856309<br>NCT02019472
Clazakizumab	anti-IL-6 moAb	RA	II	NCT02015520
Olokizumab	anti-IL-6 moAb	RA	III	NCT02760433<br>NCT02760368<br>NCT03120949
Anifrolumab	MoAb, Anti-IFN-α receptor	SSc	III	NCT05925803
		RA	II	NCT01438489
**Drugs targeting the plasmacytoid dendritic cells (pDCs)**				
Litifilimab	Anti-BDCA2 (Blood dendritic cell antigen 2) moAb	SLE	II	NCT02847598
Daxdilimab	Anti-Immunoglobulin-Like Transcript (ILT)-7 moAb, which specifically induces the depletion of pDCs	SLE	II	NCT04925934

Table S3. Common biologic agents used to treat SLE, SSc, and RA

Agent	Action, Cells	Lupus	Sclerosis	RA
Rituximab	Chimeric anti-CD20 moAb. Eliminates B cells, and a subset of T cells	Phase IV	Phase III	Phase IV<br>Approved by FDA in 2006
Abatacept	Protein, CTLA4-Ig construct. CD80/86 costimulation inhibitor, selectively modulates a key co-stimulatory signal for the full activation of T lymphocytes expressing CD28. Inhibits activated Tcells, and antigen-presenting cells.	Phase III	Phase II	Phase IV<br>Approved by FDA in 2005

Anifrolumab	MoAb, Anti-IFN-α receptor	Phase III Approved by FDA in 2021	Phase III	Phase II
Tocilizumab	moAb against IL-6R. Inhibits B cell differentiation, M2 macrophages, TH17 polarization, and myofibroblast activation	Phase I	Phase III	Phase IV Approved by FDA in 2010
